# Mobile Health Management Platform–Based Pulmonary Rehabilitation for Patients With Non–Small Cell Lung Cancer: Prospective Clinical Trial

**DOI:** 10.2196/12645

**Published:** 2019-06-21

**Authors:** Wonjun Ji, Hee Kwon, Sungin Lee, Seulgi Kim, Jeong Sook Hong, Yu Rang Park, Hyeong Ryul Kim, Jae Cheol Lee, Eun Ji Jung, Donghyun Kim, Chang-Min Choi

**Affiliations:** 1 Department of Pulmonary and Critical Care Medicine Asan Medical Center University of Ulsan College of Medicine Seoul Republic of Korea; 2 LifeSemantics Seoul Republic of Korea; 3 Department of Biomedical Systems Informatics Yonsei University College of Medicine Seoul Republic of Korea; 4 Department of Oncology Asan Medical Center University of Ulsan College of Medicine Seoul Republic of Korea

**Keywords:** mHealth, pulmonary rehabilitation, lung cancer, telemedicine, telerehabilitation, carcinoma, non-small-cell lung

## Abstract

**Background:**

Lung cancer patients experience various symptoms during treatment. Although pulmonary rehabilitation is an effective way to improve these symptoms, a medical environment of limited availability makes it difficult to provide seamless and adequate rehabilitation for lung cancer patients.

**Objective:**

This study aimed to investigate the effects of a personalized pulmonary rehabilitation program using real-time mobile patient health data for patients with non–small cell lung cancer.

**Methods:**

We conducted a prospective clinical trial in 64 patients with non–small cell lung cancer aged between 20 and 80 years at a large tertiary hospital in Seoul, South Korea. A 12-week personalized pulmonary rehabilitation program, called efil breath, was administered to determine the effectiveness of the newly developed rehabilitation app. Participants were randomly allocated to the fixed exercise or fixed-interactive exercise group (which received the personalized program). We measured changes in 6-minute walk distance (6MWD) and dyspnea (modified Medical Research Council [mMRC] score) at 6 weeks; and quality of life and service satisfaction at 12 weeks. We used the paired *t* test to analyze the variables.

**Results:**

Patients used the newly developed mobile health pulmonary rehabilitation app and a real-time patient monitoring website. In all participants, significant changes were observed in 6MWD at 12 weeks from a mean of 433.43m (SD 65.60) to 471.25m (SD 75.69; *P*=.001), and mMRC from a mean score of 0.94 (0.66) to 0.61 (SD 0.82; *P*=.02). The intervention significantly improved their quality of life (EuroQol-visual analog scale [EQ-VAS]) compared with baseline (mean score 76.05, SD 12.37 vs 82.09, SD 13.67, respectively; *P*=.002).

**Conclusions:**

A personalized mobile health–based pulmonary rehabilitation app for recording and monitoring real-time health data of patients with non–small cell lung cancer can supplement traditional health care center–based rehabilitation programs. This technology can encourage improvement of physical activity, dyspnea, and quality of life.

## Introduction

### Background

Lung cancer is a leading cause of death from cancer worldwide [1], with high rates of morbidity and mortality, as well as having a high burden of symptoms, such as dyspnea, fatigue, anxiety, depression, and pain [2,3]. These symptoms persist after diagnosis [4] but occur even in early stages of the disease [5]. A previous study showed that the survival of patients with lung cancer is improved when these symptoms are effectively managed [6]. Therefore, it is very important to provide appropriate palliative care for patients with lung cancer.

Pulmonary rehabilitation (PR) is a multidisciplinary, comprehensive treatment intervention for patients with chronic respiratory diseases [7]. PR has been shown to improve the lives of patients with chronic obstructive pulmonary disease [8], interstitial lung disease [9], and lung cancer. Although many studies have addressed the use of PR to reduce postoperative complications or improve postoperative outcomes among patients with lung cancer undergoing surgery [10-17], some studies have reported that PR can also improve exercise capacity, alleviate symptoms, and improve the quality of life (QoL) of patients with lung cancer who have received chemotherapy or radiation therapy [18-21]. Despite the reported effectiveness of PR, the application of PR to patients with lung cancer in real clinical practice environments is limited due to long waiting lists for rehabilitation services [22]. To overcome these limitations, studies on the effects of home-based PR on lung cancer patients have been reported [23-27], giving rise to the development of mobile technologies in health care delivery.

### Objective

The aim of this study was to examine the outcome of home-based PR regarding exercise capacity, dyspnea symptoms, and QoL in adult patients who were being treated for non–small cell lung cancer (NSCLC). To achieve this goal, we developed efil breath, a mobile health (mHealth) rehabilitation platform for patients with NSCLC that custom tailors symptom management and exercise programs to individual patient’s health conditions. The platform comprises 2 apps for pulmonary rehabilitation and a patient monitoring website. Patient compliance and progress were monitored for timely intervention by health care professionals. We conducted a prospective clinical trial to investigate the effectiveness of the platform. To our knowledge, this was the first clinical trial of an mHealth PR service for patients in South Korea with lung cancer.

## Methods

### Study Design

**Figure 1:**
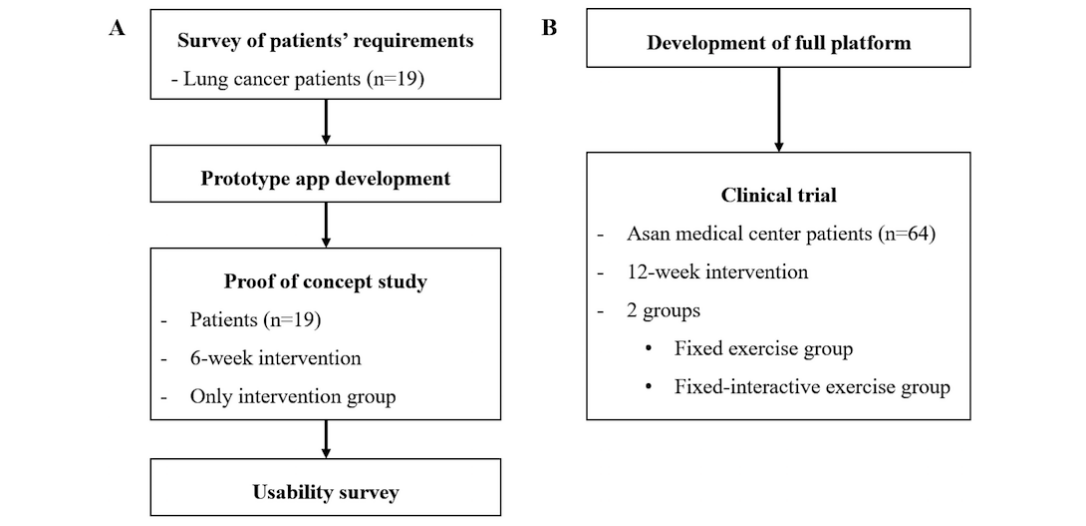
Study design. (A) Flow of the proof-of-concept study; (B) flow of the clinical trial.

We conducted the study in 2 phases: (1) a proof of concept phase, and (2) full platform development and a clinical trial (Figure 1). In the second phase, we developed 2 apps: one with a fixed exercise program, and the other with interactive exercise programs that conformed to the individual patient’s physical capacities. We also developed a patient monitoring website to collect data from the apps in real time. Although the main goal of the clinical trial was to evaluate the effects of home-based PR in the entire study population, we also evaluated the effect of PR according to the 2 apps.

### Development of a Personalized Mobile Health–Based Pulmonary Rehabilitation Platform

#### Apps

**Figure 2:**
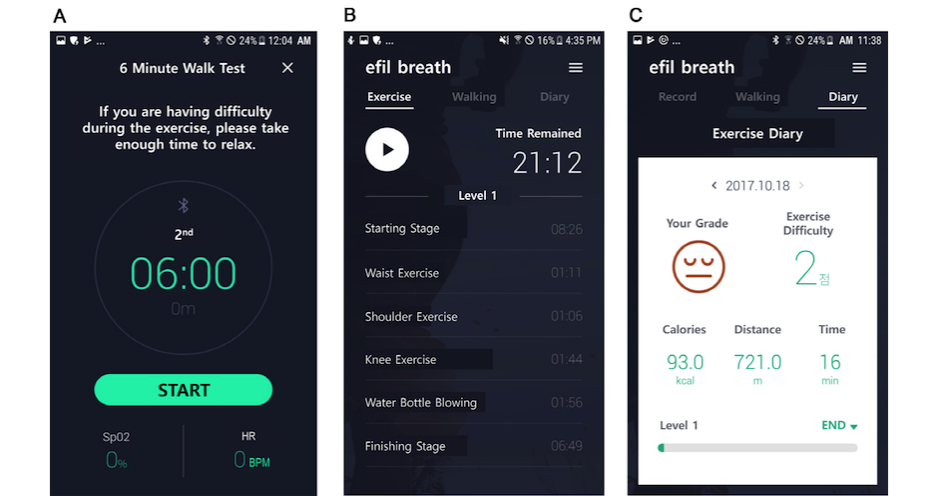
App screenshots showing (A) the 6-minute walk test, (B) exercise routines, and (C) a summary of the results.

**Figure 3:**
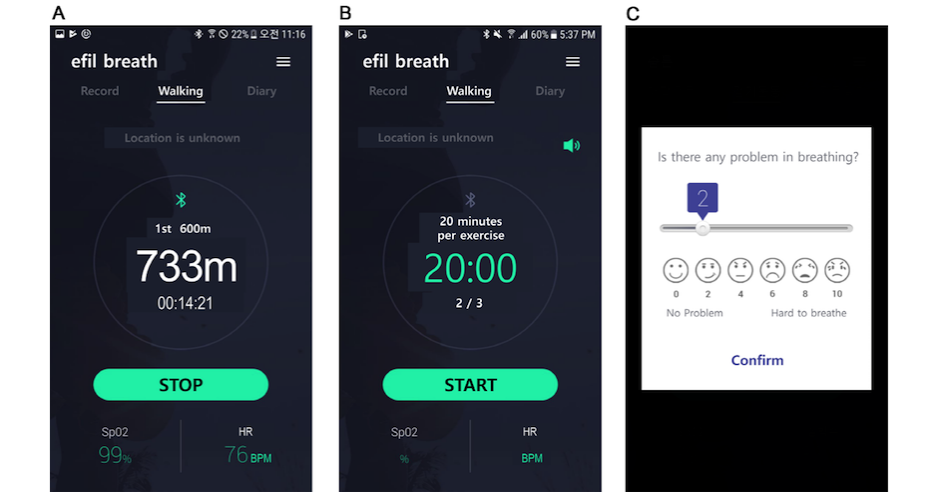
App screenshots for the walking exercise showing (A) the operation screen, (B) standby screen, and (C) measurement of dyspnea.

Use of the apps and patient monitoring website within the mHealth rehabilitation platform are described elsewhere [28]. The platform was developed as a comprehensive rehabilitation-focused platform for patients with chronic obstructive pulmonary disease [28], lung cancer, breast cancer, and others. Figures 2 and 3 show screenshots of the app for the fixed exercise program. The contents of the app were translated from Korean to English by HK.

#### Patient Monitoring Website

**Figure 4:**
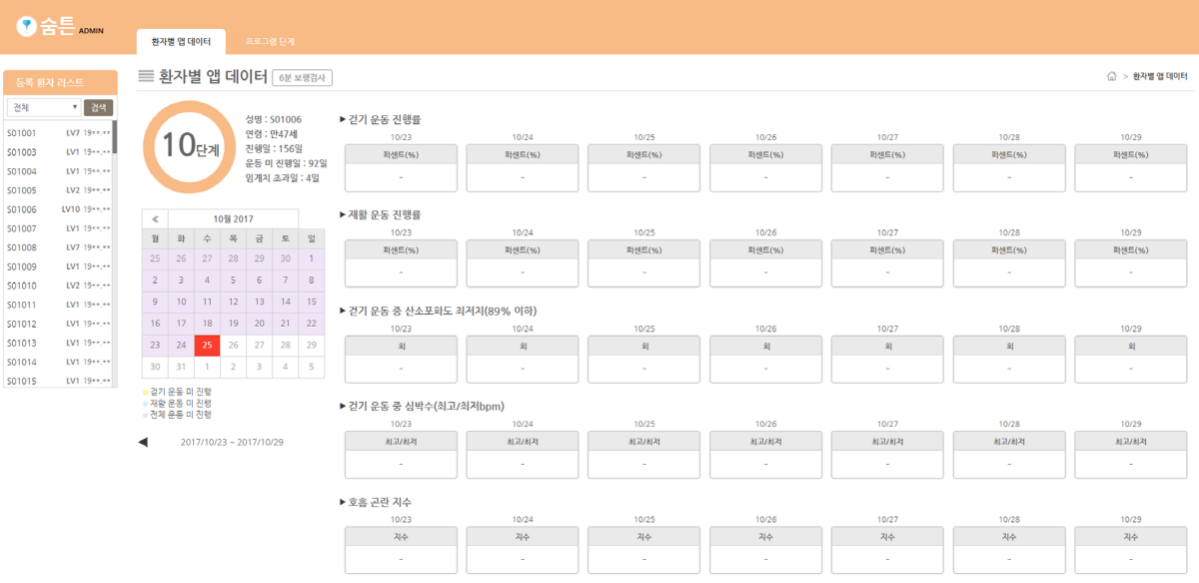
Patient health data screen showing detailed information about exercise and health measurements for a fictional patient.

All patient-generated health data, such as 6-minute walk test (6MWT) results, rehabilitation exercise progress, heart rate, and breathing difficulty levels, are sent from the apps to a central database. Health care professionals, such as lung cancer specialists and nurses, can access the database through the monitoring website but are permitted to view only the patients’ data. When an individual patient is selected, the patient’s health status is displayed, as Figure 4 shows.

### Clinical Trial

We recruited participants from May 2017 to December 2017 from the outpatient clinics of Asan Medical Center, a tertiary hospital in South Korea. This study was approved by the institutional review board of Asan Medical Center. The NSCLC participant inclusion criteria were as follows: (1) adults aged 20 to 80 years, (2) patients with histologically confirmed NSCLC, (3) the presence of obstructive ventilatory disturbance, defined by a forced expiratory volume in the first second of inspiration of less than 80% in spirometry, (4) the ability to walk more than 150 m in a 6MWT, (5) possession of an Android mobile phone, and (6) patients who agreed to provide informed written consent before the study. Patients who met the following criteria were excluded: (1) having severe cerebrovascular or musculoskeletal disease and being unable to follow the exercise regimen, (2) being illiterate or having limited communication ability, (3) having a major disability that can cause death within 1 year, or (4) declining to sign the consent form.

At the initial visit, all the participants were randomly allocated to 1 of 2 groups for 12 weeks: a fixed exercise group and a fixed-interactive exercise group. The fixed exercise group used only the fixed exercise program during the 12 weeks, whereas the fixed-interactive exercise group received the app with the fixed exercise regimen for the first 6 weeks and then switched to an app with an interactive exercise regimen for the remaining 6 weeks. We based this sequence on the expectation that the interactive exercise program could provide more effective personalized exercise based on the participant’s PR record for the previous 6 weeks. All participants performed a 6MWT, which is a standard indicator of physical activity in PR. We measured subjective dyspnea (modified Medical Research Council [mMRC] symptom score) at baseline (visit [V] 1), 6 weeks (V2), and 12 weeks (V3). Participants were provided a wearable pulse oximeter (Checkme O2, Viatom, China) to monitor their pulse rate and oxygen saturation during exercise. QoL was measured by the EuroQol 5 dimensions questionnaire (EQ-5D), which is a standardized instrument for measuring general health status and has been widely used in clinical studies and for routine outcome measurement of operational health care [29].

The primary end points were the pulmonary function parameters 6-minute walk distance (6MWD) and mMRC grade of dyspnea at 12 weeks. The secondary outcomes were QoL (EuroQol-visual analog scale [EQ-VAS] and EQ-5D) and participant satisfaction (Patient Global Assessment [PGA]) based on a 5-point Likert scale (1=strongly disagree, 2=disagree, 3=neither disagree nor agree, 4=agree, 5=strongly agree) measured at V3 (12 weeks). After confirming the change in outcome values in the entire study group, we also analyzed the difference between the fixed and fixed-interactive group.

### Measurement and Data Analysis

We performed all statistical analyses using SAS version 9.4 (SAS Institute Inc). Paired *t* test was used to evaluate the changes in pulmonary parameters in all participants and to compare the 2 exercise program groups. We considered *P*<.05 to be statistically significant. Based on previous studies of 6MWD improvement in related trials [12], we hypothesized that participants would have a mean improvement in 6MWD of 30 m at 12 weeks. Assuming an SD of 60, 2-sided test at an alpha level of .05, power of 80%, and dropout rate of 20%, a sample size of 60 patients (30 per group) was required for the primary analysis.

## Results

### Baseline Participant Characteristics

**Figure 5:**
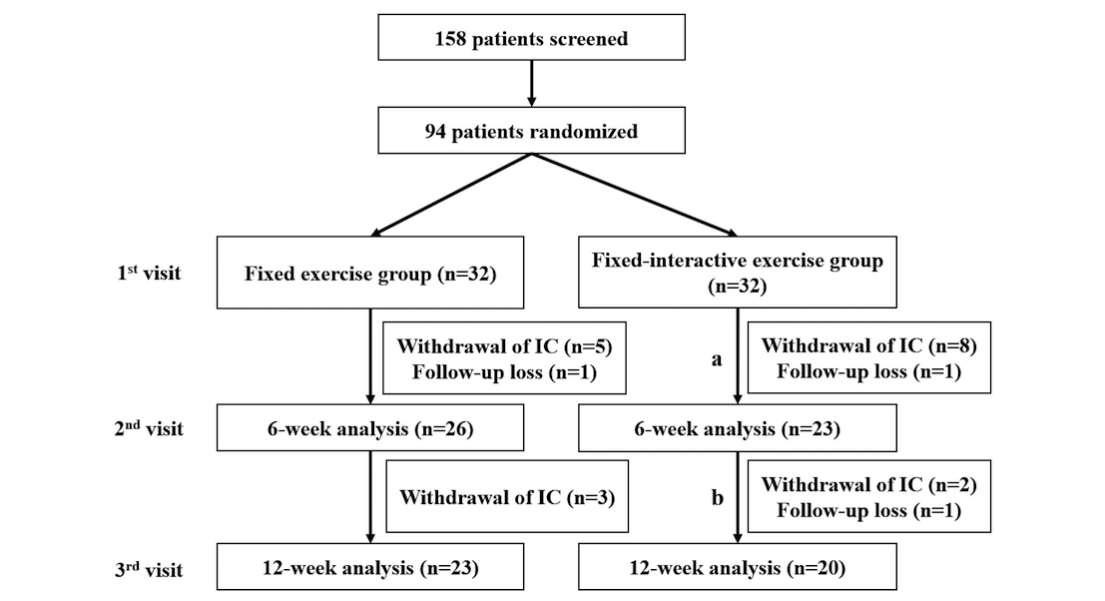
Flow of the study groups. (a) Participants using the fixed-regimen app; (b) participants using the interactive-regimen app. IC: informed consent.

We screened a total of 158 patients. Of these, 64 patients (40.5%) met the inclusion criteria and were enrolled into the 2 groups (Figure 5). Table 1 shows participants’ demographics and baseline characteristics.

**Table 1:** Baseline participant characteristics (N=64).

Variables		Fixed exercise group (n=32)		Fixed-interactive exercise group (n=32)		*P* value
		n (%)	Mean (SD)	n (%)	Mean (SD)	
Age (years)			57.97 (9.868)		60.50 (10.198)	.32
Sex: male		21 (66)		24 (75)		.41
Body mass index (kg/m^2^)			23.939 (3.332)		23.458 (3.452)	.57
**Smoking status**						.59
	Never	13 (41)		10 (31)		
	Former smoker	11 (34)		15 (47)		
	Current smoker	8 (25)		7 (22)		
**Cormobidities**						
	Diabetes mellitus	7 (22)		5 (16)		.52
	Hypertension	9 (28)		11 (34)		.59
	Cerebrovascular disease	0 (0)		0 (0)		
	Tuberculosis	4 (13)		7 (22)		.32
	Other	20 (63)		14 (44)		.27
	Previous surgery	28 (88)		21 (66)		.04
**Stage of lung cancer**						.32
	I	13 (42)		7 (22)		
	II	3 (10)		5 (16)		
	IIIA	6 (19)		7 (22)		
	IIIB	1 (3)		0 (0)		
	IV	8 (26)		13 (41)		
**Histologic type**						>.99
	Adenocarcinoma	27 (84)		27 (84)		
	Squamous	5 (16)		4 (13)		
	NSCLC^a^ and others	0 (0)		1 (3)		
**Baseline pulmonary function tests**						
	FEV_1_^b^ (% predicted)		71.31 (18.220)		68.19 (10.772)	.41
	FVC^c^ (% predicted)		75.03 (17.453)		74.03 (11.232)	.79
	FEV_1_ / FVC^d^ (% predicted)		0.96 (0.150)		0.93 (0.112)	.37
	6-minute walk distance (m)		433.13 (55.790)		427.13 (79.034)	.73
**Modified Medical Research Council dyspnea scale score**						.07
	0	11 (35)		3 (10)		
	1	17 (53)		25 (78)		
	2	3 (10)		2 (6)		
	3	1 (3)		2 (6)		
EQ-5D^e^			7.50 (1.951)		7.50 (2.356)	>.99

^a^NSCLC: non–small cell lung cancer.

^b^FEV_1_: forced expiratory volume in the first second of inspiration; normal value ≥80%.

^c^FVC: forced vital capacity; normal value ≥80%.

^d^FEV_1_ / FVC: normal value ≥70%.

^e^EQ-5D: EuroQol 5 dimensions questionnaire.

### Comparison Between Preintervention and Postintervention Changes in Primary and Secondary Outcomes by Participant

For all participants in both groups, the 6MWD improved significantly from V1 to V3 (from mean 433.43 m [SD 65.60] to 471.25m [SD 75.69]; *P*=.001). Similarly, mMRC score improved from V1 to V3 (from 0.94, SD 0.66 to 0.61, SD 0.82; *P*=.02; Table 2). As a measure of QoL, EQ-VAS score improved significantly from 76.05 (SD 12.37) at V1 to 82.09, (SD 13.67) at V3 (*P*=.002). The mean value of EQ-5D was not significantly different between time points (Table 2). PGA scores measured at V3 showed significant improvement over PGA scores at V2 (from 13.77, SD 3.68 to 15.08, SD 3.99; *P*=.01).

**Table 2:** Results of all participants’ outcome variables according to time point.

Variables	Visit 1 (baseline, N=49)		Visit 2 (6 weeks, N=49)			Visit 3 (12 weeks, N=43)		
	n (%)	mean (SD)	n (%)	mean (SD)	*P* value	n (%)	mean (SD)	*P* value
6MWD^a^ (m)	49 (100)	433.429 (65.595)	42 (86)	448.095 (76.421)	.14	40 (93)	471.250 (75.691)	.001
mMRC^b^ score	49 (100)	0.939 (0.659)	45 (92)	0.733 (0.618)	.04	43 (100)	0.605 (0.821)	.02
EQ-VAS^c^ score	43 (88)	76.047 (12.371)	N/A^d^	N/A	N/A	43 (100)	82.093 (13.674)	.002
EQ-5D^e^ score	43 (88)	7.535 (1.817)	N/A	N/A	N/A	43 (100)	6.930 (2.849)	.17
PGA^f^ score	N/A	N/A	39 (80)	13.769 (3.681)	N/A	39 (91)	15.077 (3.989)	.01

^a^6MWD: 6-minute walk distance.

^b^mMRC: modified Medical Research Council.

^c^EQ-VAS: EuroQol-visual analog scale.

^d^N/A: not applicable.

^e^EQ-5D: EuroQol 5 dimensions questionnaire.

^f^PGA: Patient Global Assessment.

**Table 3:** Preintervention and postintervention changes in outcome variables between the 2 groups.

Variables	Fixed exercise group (N=23)		Fixed-interactive exercise group (N=20)		*P* value
	n (%)	Mean (SD)	n (%)	Mean (SD)	
6MWD^a^ (m)	21 (91)	58.095 (73.663)	19 (95)	25.368 (66.640)	.30
mMRC^b^ score	23 (100)	–0.435 (0.945)	20 (100)	–0.250 (0.910)	.68
EQ-VAS^c^ score	23 (100)	6.304 (9.073)	20 (100)	5.750 (14.825)	.99
EQ-5D^d^ score	23 (100)	–0.957 (1.745)	20 (100)	–0.200 (3.722)	.50

^a^6MWD: 6-minute walk distance.

^b^mMRC: modified Medical Research Council.

^c^EQ-VAS: EuroQol-visual analog scale.

^d^EQ-5D: EuroQol 5 dimensions questionnaire.

### Comparison Between Preintervention and Postintervention Changes in Primary and Secondary Outcomes by Exercise Program

Unlike the results of all participants, there were no statistical differences in the primary and secondary outcome indicators between the fixed and fixed-interactive exercise groups (Table 3).

## Discussion

### Principal Findings

We developed a personalized mHealth PR platform to provide rehabilitation treatment for patients with lung cancer. Our mobile app-based PR improved exercise capacity (as measured by 6MWD), dyspnea grade, and QoL. This suggests that a mobile-based home rehabilitation program might be an alternative method of conventional PR for patients with lung cancer, especially under the limitations of the clinical environment in South Korea, where many PR programs are inadequate. We demonstrated that personalized PR using mobile technology significantly improved exercise capacity and QoL for patients with lung cancer regardless of disease status.

Previous studies that reported the effects of PR in patients with lung cancer focused mostly on perioperative periods [10-17,23], whereas only a few studies assessed patients with advanced lung cancer [19,21,24,27,30]. In this study, we analyzed the effect of PR on both early- and advanced-stage lung cancer patients, and found improvements in exercise capacity (6MWD) and dyspnea, consistent with previous studies that reported improvements in exercise capacity [10-13,16,21,24,27] or dyspnea grade [21,27].

Only a few studies have reported on the effect of PR on the QoL of patients with lung cancer [11,24,26,27], and evidence is still insufficient. Although Stigt et al [11] reported that PR did not improve patients’ QoL after lung cancer surgery, other studies supported the positive effect of PR [26,27]. Our study found that EQ-VAS was significantly improved at the end of PR, compared with baseline, which is consistent with previous studies [24] reporting significant improvement in daily physical activity and anxiety scores. However, we did not find a significant improvement in EQ-5D, which measures QoL in 5 dimensions: mobility, self-care, daily activity, pain/discomfort, and anxiety/depression. Each item is scored from 1 to 5; the higher the score, the lower the QoL. On the other hand, EQ-VAS scores reflect patients’ subjective perception of QoL on a continuous scale from 0 to 100 points. A score of 100 indicates the best health condition, whereas a score of 0 indicates the worst condition. In our study, the EQ-VAS scores showed a more widely distributed pattern than the EQ-5D scores; we suggest that this difference could be responsible for the difference in the measures’ statistical significance. Importantly, these 2 indicators have consistently shown that PR can improve the QoL of patients with lung cancer.

### Limitations

This study had a few limitations. First, the proportion of patients with advanced lung cancer was relatively small. About 70% of the patients in this study had surgical treatments prior to PR, and about 33% were in an advanced stage of the disease. Considering that lung cancer is diagnosed at advanced stages in about two-thirds of patients [1], further studies involving more patients with advanced-stage lung cancer are needed to better confirm the effects of mobile-based PR.

Second, this study reported the effects of 12-week PR on patients with lung cancer, but it did not assess long-term effects. Most studies of PR for patients with lung cancer have reported only a 12-week follow-up, similar to this study. Future studies are required to confirm the long-term benefit of PR platforms for both patients and health care professionals. Also, this mobile-based personalized rehabilitation platform should be studied in patients with lung cancer who live in medically deprived areas, such as remote islands with small populations.

Third, only 40.5% of the screened patients were enrolled in the study. As Table 1 shows, the participants’ mean age was around 60 years; thus, many patients had difficulty with handling the technology or did not own an adequate device for this study. Some patients declined to use this app-based PR service. Together, these suggest that technology limitations might affect the application of home-based PR service among the elderly. Therefore, further research is warranted to study how to best provide effective PR service using smart devices to older patients.

Fourth, we found no significant difference between the fixed and the fixed-interactive exercise regimen groups. To our knowledge, this was the first clinical study to confirm the effectiveness of home-based PR using app-based exercise programs in patients with lung cancer. Although the main goal of this study was to evaluate the effects of home-based PR, we expected that the interactive exercise program could provide more effective personalized exercise based on the participant’s PR record for the previous 6 weeks. We suggest that the 6-week duration was likely too short to create a meaningful difference between the 2 groups. Further prospective studies are needed to evaluate the effects of long-term exercise according to whether participants followed a fixed or fixed-interactive exercise program.

### Conclusions

Personalized mHealth-based PR in patients with NSCLC can supplement traditional health care center–based rehabilitation programs. Our clinical trial results support the use of this technology to improve exercise capacity, dyspnea symptoms, and QoL. mHealth technology is now a robust and supplementary tool for self-management, remote monitoring, and rehabilitation for patients who require chronic care. The relevance of mHealth-based rehabilitation is enhanced only when its rehabilitative efficacy is corroborated by evidence-based clinical results. Evidence-based PR for lung cancer enables prescription of exercise regimens commensurate with a patient’s individual physical status. To our knowledge, efil breath is the first attempt in South Korea at developing an mHealth management platform for patients with lung cancer. The results of this study may form the foundation of other mHealth-based PR endeavors to support optimal home-based self-management.

## Abbreviations

6MWD: 6-minute walk distance
6MWT: 6-minute walk test
EQ-5D: EuroQol 5 dimensions questionnaire
EQ-VAS: EuroQol-visual analog scale
mHealth: mobile health
mMRC: modified Medical Research Council
NSCLC: non–small cell lung cancer
PGA: Patient Global Assessment
PR: pulmonary rehabilitation
QoL: quality of life
V: visit
